# Mitotic slippage is determined by p31^comet^ and the weakening of the spindle-assembly checkpoint

**DOI:** 10.1038/s41388-020-1187-6

**Published:** 2020-02-06

**Authors:** Tsun Ming Lok, Yang Wang, Wendy Kaichun Xu, Siwei Xie, Hoi Tang Ma, Randy Y. C. Poon

**Affiliations:** 1 Division of Life Science, Center for Cancer Research, and State Key Laboratory of Molecular Neuroscience, Hong Kong University of Science and Technology, Clear Water Bay, Hong Kong; 2 Present Address: Department of Molecular Biosciences, Institute for Cellular and Molecular Biology, The University of Texas at Austin, Austin, TX USA

**Keywords:** Mitosis, Chromosomes

## Abstract

Mitotic slippage involves cells exiting mitosis without proper chromosome segregation. Although degradation of cyclin B1 during prolonged mitotic arrest is believed to trigger mitotic slippage, its upstream regulation remains obscure. Whether mitotic slippage is caused by APC/C^CDC20^ activity that is able to escape spindle-assembly checkpoint (SAC)-mediated inhibition, or is actively promoted by a change in SAC activity remains an outstanding issue. We found that a major culprit for mitotic slippage involves reduction of MAD2 at the kinetochores, resulting in a progressive weakening of SAC during mitotic arrest. A further level of control of the timing of mitotic slippage is through p31^comet^-mediated suppression of MAD2 activation. The loss of kinetochore MAD2 was dependent on APC/C^CDC20^, indicating a feedback control of APC/C to SAC during prolonged mitotic arrest. The gradual weakening of SAC during mitotic arrest enables APC/C^CDC20^ to degrade cyclin B1, cumulating in the cell exiting mitosis by mitotic slippage.

## Introduction

Nearly the entire cell physiological environment is reorganized during mitosis to facilitate division. When mitosis is completed, all the cellular changes are reversed to return the daughter cells to interphase. Cyclin-dependent kinase 1 (CDK1) and its activating subunit cyclin B1 are essential components of the mitotic engine. Consequently, the destruction of cyclin B1, enforced by a ubiquitin ligase comprised of anaphase-promoting complex/cyclosome and its targeting subunit CDC20 (APC/C^CDC20^), is a key event triggering mitotic exit [1]. During early mitosis, APC/C^CDC20^ is inhibited by the spindle-assembly checkpoint (SAC), which senses unattached or improperly attached kinetochores [2]. This ensures that APC/C^CDC20^ activation, and thus mitotic exit, only occurs after all the chromosomes have achieved proper bipolar spindle attachment.

Activation of SAC is initiated by MAD1–MAD2 complexes at kinetochores, which then serve as templates for converting other MAD2 from an open conformation (O-MAD2) to a closed conformation (C-MAD2) [3]. Upon this structural remodeling, the C-terminal CDC20-binding site of MAD2 is exposed to enable it to interact with CDC20. The C-MAD2 then forms a diffusible mitotic checkpoint complex (MCC) comprising of MAD2, BUBR1, BUB3, and CDC20, which binds APC/C^CDC20^ (containing a second CDC20) and suppresses its activity. After SAC is satisfied, new C-MAD2 is no longer generated from the kinetochores. The existing C-MAD2 is converted to O-MAD2 by a process involving p31^comet^ and TRIP13 [4–7]. This releases APC/C^CDC20^ from inhibition by the SAC, allowing the cell to exit mitosis.

As APC/C^CDC20^ is active only after SAC is satisfied, agents that disrupt spindle dynamics can trigger a prolonged mitotic arrest [8]. Classic examples include spindle poisons that attenuate microtubule depolymerization or polymerization (e.g., taxanes and vinca alkaloid, respectively). Nevertheless, the fate of individual cells after protracted mitotic arrest varies greatly [9]. On the one hand, the accumulation of apoptotic activators and/or a loss of apoptotic inhibitors during mitotic arrest can induce mitotic cell death. On the other hand, cells can exit mitosis without proper chromosome segregation and cytokinesis in a process termed mitotic slippage. The current paradigm states that an underlying mechanism of mitotic slippage is a gradual degradation of cyclin B1 during mitotic arrest [10]. In support of this, cells lacking APC/C^CDC20^ activity are unable to undergo mitotic slippage [11].

Although the prevailing view is that degradation of cyclin B1 plays a critical role in mitotic slippage, it is probably too simplistic a view. Why cyclin B1 can be degraded in the presence of an active SAC? What is the origin of the signal for cyclin B1 degradation? One hypothesis is that the leakage of cyclin B1 degradation is caused by a low-APC/C^CDC20^ activity that is able to escape SAC-mediated inhibition. An alternative hypothesis is that cyclin B1 degradation is due to a gradual weakening of SAC, caused by a fatigue in SAC activation and/or strengthening of SAC-inactivating mechanisms. In this study, we found that reduction of MAD2 at the kinetochores during mitotic arrest initiates a weakening of the SAC, thereby enabling APC/C^CDC20^ to degrade cyclin B1 in a proteasome-dependent manner to promote mitotic slippage.

## Results

### Shifting mitotic cell fates to APC/C^CDC20^-dependent mitotic slippage in HeLa cells

**Fig. 1:**
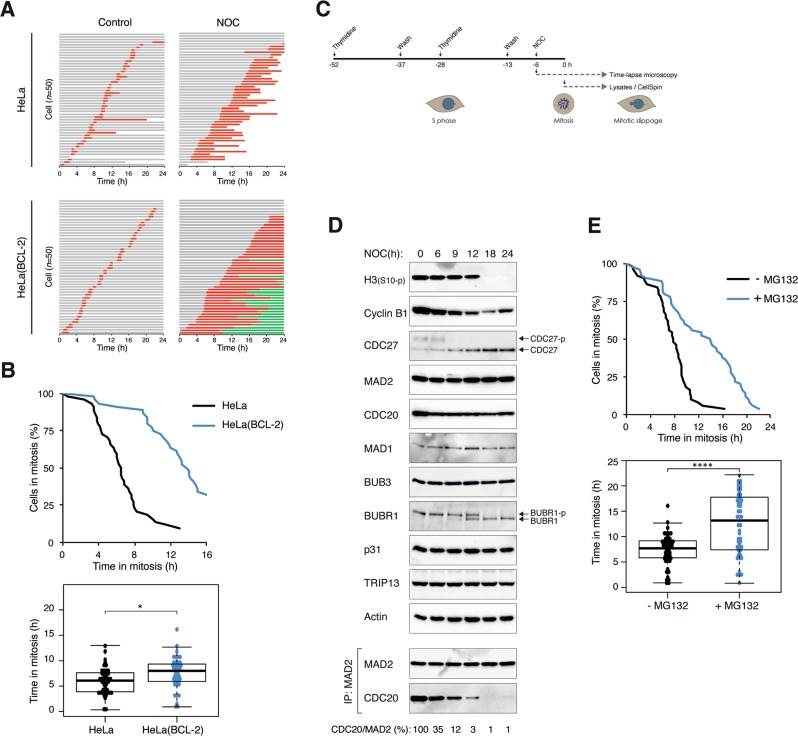
Inhibition of apoptosis promotes proteasome-dependent mitotic slippage. **a** Inhibition of apoptosis promotes mitotic slippage. HeLa or HeLa expressing BCL-2 were incubated with NOC. Individual cells were then tracked using live-cell imaging for 24 h. Key: interphase (gray); mitosis (red); interphase after mitotic slippage (green); truncated bars (cell death). **b** Inhibition of apoptosis extends the duration of mitotic arrest. The duration of mitotic arrest of cells from **a** is plotted by using Kaplan–Meier estimator. Box-and-whisker plots show the elapsed time between mitotic entry and mitotic cell death/exit. Note that cells that were still in mitosis at the end of the imaging were also included in the statistical analysis. **c** Schematic diagram of the synchronization procedure to obtain cells arrested in mitosis. HeLa cells expressing BCL-2 were synchronized at G_1_/S with a double-thymidine procedure, and released into the cell cycle before treatment with NOC. The cells were then monitored using live-cell imaging to analyze cell fate at single cells level. Alternatively, mitotic cells were isolated by mechanical shake off, continued to be incubated with NOC, and harvested at different time points for biochemical analyses. **d** Mitotic markers, SAC components, and MCC during mitotic slippage. Cells were synchronized and treated with NOC as described in **c**. Lysates were prepared from cells harvested at the indicated time points and subjected to immunoprecipitation (IP) using antiserum against MAD2. The immunoprecipitates and total lysates were then analyzed using immunoblotting. The positions of the phosphorylated and unphosphorylated forms of BUBR1 and CDC27 are indicated. Uniform loading of lysates was confirmed by immunoblotting for actin. The band intensities of CDC20 in the MAD2 immunoprecipitates were quantified, normalized with MAD2, and expressed as % max. **e** Mitotic slippage is delayed after inhibition of the proteasome. HeLa cells expressing both BCL-2 and histone H2B-GFP were synchronized and treated with NOC as described in **c**. The cells were incubated with either buffer or MG132. Individual cells were then tracked using live-cell imaging. The duration of mitotic arrest and the elapsed time between mitotic entry and mitotic cell death/slippage are shown. The live-cell imaging profile of individual cells is shown in Fig. S1C.

Due to its relatively slow intrinsic mitotic slippage rate compared with many cancer cell lines, HeLa was used as a model for studying events leading to mitotic slippage induced by the spindle poison nocodazole (NOC). The antiapoptotic protein BCL-2 was overexpressed in these cells to uncouple mitotic cell death, as indicated by the reduction of PARP1 cleavage (Fig. S1A) and sub-G_1_ population (Fig. S1B). The presence of cells possessing DNA contents higher than G_2_/M indicated that the increase in survival was accompanied by mitotic slippage and DNA rereplication (Fig. S1B). Live-cell imaging analysis further confirmed that expression of BCL-2 was able to switch cell fate from apoptosis to mitotic slippage (Fig. 1a). The inhibition of mitotic cell death was accompanied with a longer mitotic delay before cells either underwent mitotic slippage or reached the end of the imaging period (Fig. 1b).

To prevent individual cells from exposing to NOC for different amount of time before mitosis, cells were first synchronized with a double-thymidine procedure before incubated with NOC (Fig. 1c). Mitotic cells were isolated by mechanical shake off (*t* = 0) and continued to be incubated in NOC-containing medium. The gradual loss of cyclin B1 is consistent with its role in mitotic slippage, which was confirmed by the loss of mitotic markers including phosphorylated histone H3^Ser10^ and CDC27 (Fig. 1d). In agreement, cyclin B1 degradation (see later) and mitotic slippage (Figs. 1e and S1C) were delayed in the presence of the proteasome inhibitor MG132.

**Fig. 2:**
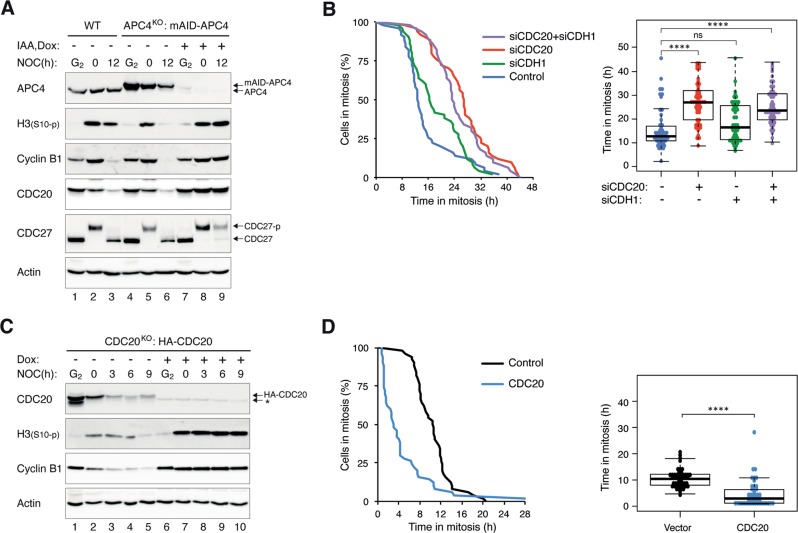
Mitotic slippage requires APC/C^CDC20^. **a** Depletion of APC4 prevents mitotic slippage. HeLa (WT) or APC4^KO^ expressing mAID-APC4 cells were synchronized as described in Fig. 1c with minor modifications: IAA and Dox were added (at 15 h after second thymidine release) to turn off mAID-APC4. The cells were harvested either at G_2_ (22 h after second thymidine release) or at different time points in mitosis. Lysates were prepared and the indicated proteins were detected with immunoblotting. **b** Downregulation of CDC20 delays mitotic slippage. HeLa cells were transfected with siRNA against CDC20 and/or CDH1. The cells were treated with NOC as described in Fig. 1c (transfection was performed at first thymidine release) before analyzed using live-cell imaging. The duration of mitotic arrest and the elapsed time between mitotic entry and mitotic cell death/slippage are shown. The immunoblotting or CDC20 and CDH1 and the live-cell imaging profile of individual cells are shown in Fig. S2A. **c** Conditional depletion of CDC20 delays mitotic slippage. CDC20^KO^ cells expressing HA-CDC20 were synchronized as described in Fig. 1c with minor modifications: Dox was added to turn off the expression of HA-CDC20 (at 14 h after first thymidine release). The cells were harvested either at G_2_ (22 h after second thymidine release) or at different time points in mitosis. Lysates were prepared and the indicated proteins were detected with immunoblotting. The asterisk indicates the position of a cross-reactive band by the CDC20 antibodies (see Fig. 7e). **d** Overexpression of CDC20 promotes mitotic slippage. Cells were transfected with either vector or a plasmid expressing FLAG-CDC20. A plasmid expressing ECFP was co-transfected. After 40 h, the cells were treated with NOC and analyzed using live-cell imaging. The duration of mitotic arrest and the elapsed time between mitotic entry and mitotic cell death/slippage are shown. The live-cell imaging profile of individual cells and CDC20 expression are shown in Fig. S2C.

We next monitored the activity of APC/C during mitotic arrest using the reporter system fluorescent ubiquitin-based cell-cycle indicators (FUCCI) [12]. The signal intensity of the APC/C reporter progressively decreased during mitotic arrest, indicating that a subset of the APC/C was activated during mitotic arrest (Fig. S1D). In line with APC/C-dependent degradation, the decrease of the reporter could be inhibited with MG132. More direct evidence of the role of APC/C in mitotic slippage was obtained by depleting the APC/C subunit APC4 using CRISPR–Cas9 (Fig. 2a). To generate cells with knockout (KO) of APC4 (an essential gene), we first expressed a mini-auxin-inducible degron (mAID)-tagged APC4, which can be degraded rapidly in response to indole-3-acetic acid (IAA) in cells expressing the ubiquitin ligase SCF^TIR1^ [13] before disrupting the endogenous APC4. Consequently, APC4 could be conditionally inactivated in the presence of IAA and doxycycline (Dox) (the mAID-APC4 was also under the control of a Tet-Off promoter). Figure 2a shows that conditional inactivation of APC4 prevented the destruction of APC/C targets including cyclin B1 and CDC20 during mitotic arrest and the consequent mitotic slippage (indicated by the phosphorylation of histone H3^Ser10^ and CDC27).

To determine which of the APC/C targeting subunits participate in mitotic slippage, CDC20, and/or CDH1 were depleted using siRNAs. Mitotic slippage was delayed after the knockdown of CDC20, but not CDH1 in HeLa cells (Figs. 2b and S2A). This was further confirmed using another cell line (H1299; Fig. S2B). As siCDC20 could not completely deplete CDC20, we further demonstrated the contribution of CDC20 in mitotic slippage by generating a CDC20^KO^ cell line expressing HA-CDC20 under the control of an inducible promoter. Figure 2c shows that cyclin B1 was stabilized, and mitotic slippage was inhibited after CDC20 was conditionally depleted.

Finally, we performed the converse experiment by overexpressing CDC20. Figure 2d shows that mitotic slippage was accelerated in the presence of ectopically expressed CDC20. Collectively, these data indicate that after apoptosis was abolished, the timing of mitotic slippage is controlled by APC/C^CDC20^ but not APC/C^CDH1^.

### Progressive weakening of SAC underlies the activation of APC/C^CDC20^ during mitotic arrest

During unperturbed mitosis, premature mitotic exit is prevented due to the inhibition of APC/C^CDC20^ by SAC. We next investigated if the APC/C^CDC20^ activity observed during mitotic arrest is also regulated by SAC. Although the expression of individual SAC components remained constant during mitotic arrest (MAD1, MAD2, BUB3, BUBR1, CDC20, p31^comet^, and TRIP13), the abundance of MAD2–CDC20 complexes decreased progressively (Fig. 1d). This was also reflected in the decrease in MAD2–CDC20 binding to APC/C (see later). Notably, the MAD2–CDC20 complexes started to dissociate before the onset of mitotic slippage.

**Fig. 3:**
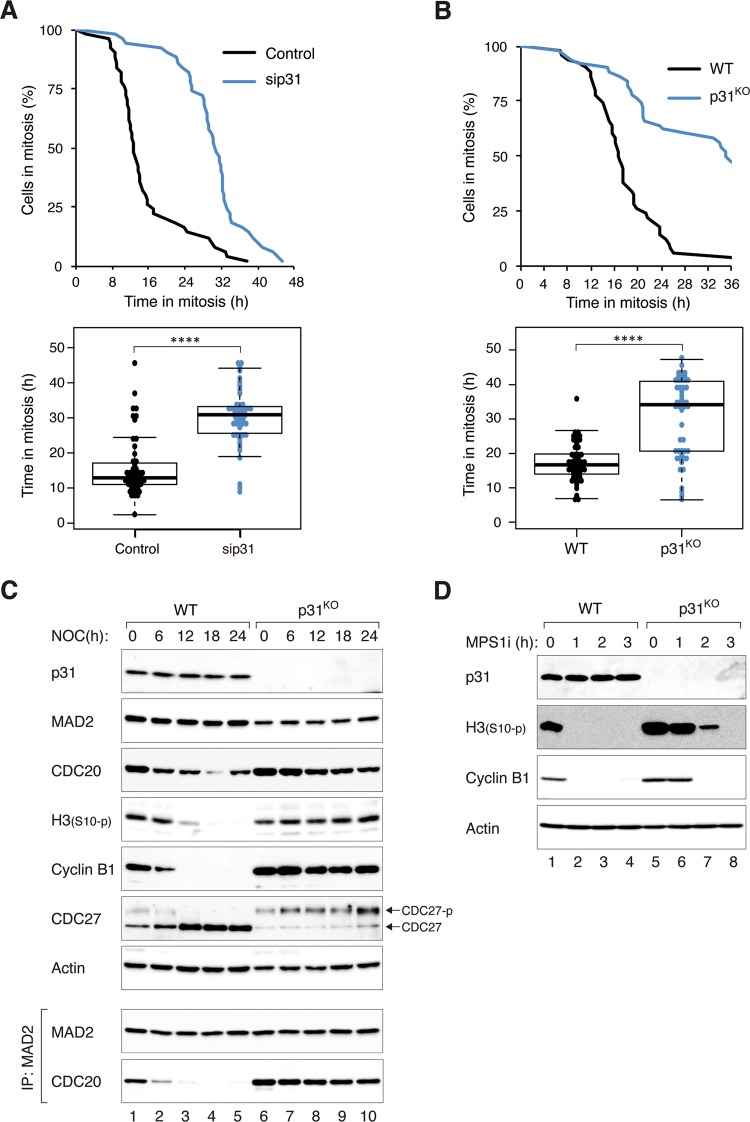
p31^comet^ controls the timing of mitotic slippage by regulating MCC. **a** Mitotic slippage is delayed after knockdown of p31^comet^. HeLa cells expressing BCL-2 were transfected with either control or siRNA against p31^comet^ (sip31). Knockdown of p31^comet^ was confirmed with immunoblotting (Fig. S3A). The cells were synchronized and treated with NOC as described in Fig. 1c. Individual cells were then tracked using live-cell imaging. The duration of mitotic arrest and the elapsed time between mitotic entry and mitotic cell death/slippage are shown. The live-cell imaging profile of individual cells is shown in Fig. S3B. **b** Mitotic slippage is delayed in p31^comet^-deficient cells. HeLa (WT) and p31^comet^-deficient cells (p31^KO^) (both stably expressing BCL-2) were incubated with NOC before analyzed using live-cell imaging. The duration of mitotic arrest and the elapsed time between mitotic entry and mitotic cell death/slippage are shown. The live-cell imaging profile of individual cells is shown in Fig. S4A. **c** MCC inactivation is delayed in the absent of p31^comet^. WT and p31^KO^ (both stably expressing BCL-2) were synchronized and treated with NOC as described in Fig. 1c. The cells were harvested at the indicated time points. Lysates were prepared and subjected to immunoprecipitation (IP) using antiserum against MAD2 followed by immunoblotting analysis. **d** Knockout of p31^comet^ delays MPS1i-mediated mitotic slippage. WT and p31^KO^ (both stably expressing BCL-2) were synchronized and treated with NOC as described in Fig. 1c. At *t* = 0 h, the cells were treated with the MPS1 inhibitor AZ 3146. Lysates were prepared at the indicated time and analyzed with immunoblotting.

The gradual loss of MAD2–CDC20 complexes before the onset of mitotic slippage suggested that it could be a cause of mitotic slippage. To test directly if SAC determines the timing of mitotic exit, the rate of mitotic slippage in the absence of p31^comet^ was analyzed. The MAD2-binding protein p31^comet^ inactivates MCC by facilitating the conversion of C-MAD2 to O-MAD2 by TRIP13 [4–7]. It is also believed that p31^comet^ binds to C-MAD2, restraining it from acting as a template to convert O-MAD2 into C-MAD2 [14, 15]. Accordingly, p31^comet^ deficiency results in a delay in MCC inactivation during unperturbed mitosis [4]. Using live-cell imaging analysis, we found that mitotic slippage was significantly delayed after p31^comet^ was downregulated with siRNA (Figs. 3a and S3A, B). Similar results were obtained using H1299, indicating that the effect of p31^comet^ on mitotic slippage is not limited to HeLa cells (Fig. S3C).

We further verified the negative regulation of mitotic slippage by p31^comet^ using p31^comet^-deficient cells (p31^KO^) (Figs. 3b and S4A). We then performed a rescue experiment by reintroducing FLAG-p31^comet^ to the p31^KO^ cells (Fig. S4B). As the expression of FLAG-p31^comet^ was slightly higher than the endogenous p31^comet^, the duration of mitotic arrest was actually shorter than in wild-type (WT) cells due to premature mitotic slippage. Protein analysis also verified the delay in mitotic slippage in the absence of p31^comet^ (Fig. 3c). Mitotic markers, including phosphorylated histone H3^Ser10^ and CDC27, were stabilized during mitotic arrest in p31^KO^ cells. Notably, the reduction of cyclin B1 was also abolished in the absence of p31^comet^. In agreement with the importance of SAC inactivation in mitotic slippage, the decrease of MAD2–CDC20 complexes during mitotic arrest was abolished in p31^KO^ cells. To confirm that mitotic slippage induced by the inactivation of SAC could be delayed by KO of p31^comet^, we next abolished SAC using an MPS1 inhibitor (MPS1i) AZ 3146. In NOC-treated WT cells, MPS1i triggered rapid mitotic slippage. By contrast, mitotic slippage was delayed in p31^KO^ cells (Fig. 3d).

Collectively, these data support the idea that mitotic slippage could be initiated by the gradual inactivation of SAC during mitotic arrest, which is promoted by p31^comet^.

### p31^comet^ accelerates mitotic slippage mainly through suppressing MAD2 activation but not TRIP13-dependent MAD2 inactivation

Conceptually, p31^comet^ can inhibit SAC by two mechanisms. First, p31^comet^ can block MAD2 activation through binding to the dimerization motif of C-MAD2, thereby competing with O-MAD2 for C-MAD2 binding [14, 15]. Second, p31^comet^ can facilitate the inactivation of existing C-MAD2 through a TRIP13-dependent mechanism [4–7]. To distinguish these possibilities, various p31^comet^ mutants that affect binding to MAD2 and/or TRIP13 were utilized (Fig. S5A, B). Ectopic expression of either WT or a mutant p31^comet^ unable to bind TRIP13 (P228A/K229A [7]; p31(PK) herein) could induce rapid mitotic slippage (Fig. S5C). By contrast, a mutant unable to bind MAD2 (Q83A/F191A [14]; p31(QF) herein) did not promote mitotic slippage. Likewise, a p31^comet^ mutant containing both QF and PK mutations did not affect mitotic slippage.

**Fig. 4:**
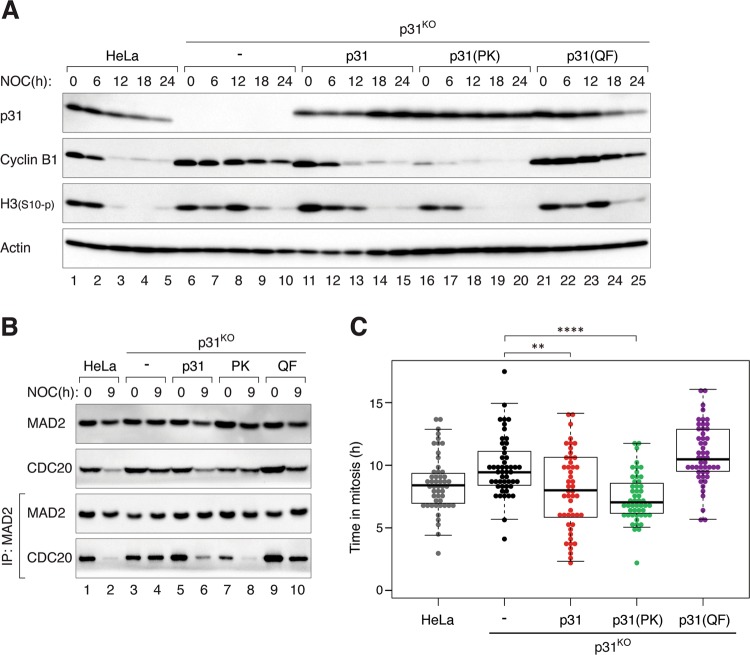
The promotion of mitotic slippage by p31^comet^ does not require binding to TRIP13. **a** Mitotic slippage in p31^KO^ cells is restored by a non-TRIP13-binding mutant. HeLa, p31^KO^, p31^KO^ expressing HA-tagged p31, p31(PK), or p31(QF) were synchronized and treated with NOC as described in Fig. 1c. Caspase inhibitors were added in this experiment to inhibit cell death. The cells were harvested at the indicated time points. Lysates were prepared and analyzed with immunoblotting. **b** Reduction of MCC during mitotic arrest is restored by a non-TRIP13-binding p31^comet^. Cells were treated with NOC as described in **a** and harvested at the indicated time points. Lysates were prepared and subjected to immunoprecipitation (IP) using antiserum against MAD2 followed by immunoblotting analysis. **c** Mitotic slippage in p31^KO^ cells is restored by a non-TRIP13-binding mutant. Cells were treated with NOC as described in **a** and analyzed using live-cell imaging. Box-and-whisker plots show the elapsed time between mitotic entry and mitotic cell death/exit (*n* = 50).

Although the above results suggested that the inactivation of existing C-MAD2 by p31^comet^–TRIP13 pathway contributes a relatively minor effect to mitotic slippage, they were based on studies with ectopic expression of p31^comet^. To express the p31^comet^ mutants at a more physiological level, we first disrupted the endogenous p31^comet^ gene (*MAD2L1BP*) before introducing different p31^comet^ constructs into the cells to obtain stable cell lines. Clones were selected for those expressing exogenous p31^comet^, p31(PK), or p31(QF) at levels comparable to the endogenous p31^comet^ (Fig. 4a). As before, KO of p31^comet^ resulted in a delay in mitotic slippage (as revealed by cyclin B1 degradation and histone H3^Ser10^ dephosphorylation). While p31^comet^ or p31(PK) restored the normal rate of mitotic slippage, p31(QF)-expressing cells underwent a delayed mitotic slippage similarly as p31^KO^ cells.

To obtain further evidence that p31^comet^ does not require TRIP13 binding to promote mitotic slippage, we also examined MAD2–CDC20 complexes during mitotic arrest (Fig. 4b). As shown above, deletion of p31^comet^ prevented the loss of MAD2–CDC20 complexes over time. This could be restored by reintroducing p31^comet^ or p31(PK), but not by p31(QF). In agreement with the levels of mitotic markers and MAD2–CDC20 complexes, live-cell imaging analysis also indicated that the delay in mitotic slippage in p31^KO^ cells was restored by p31^comet^ or p31(PK), but not by p31(QF) (Fig. 4c).

**Fig. 5:**
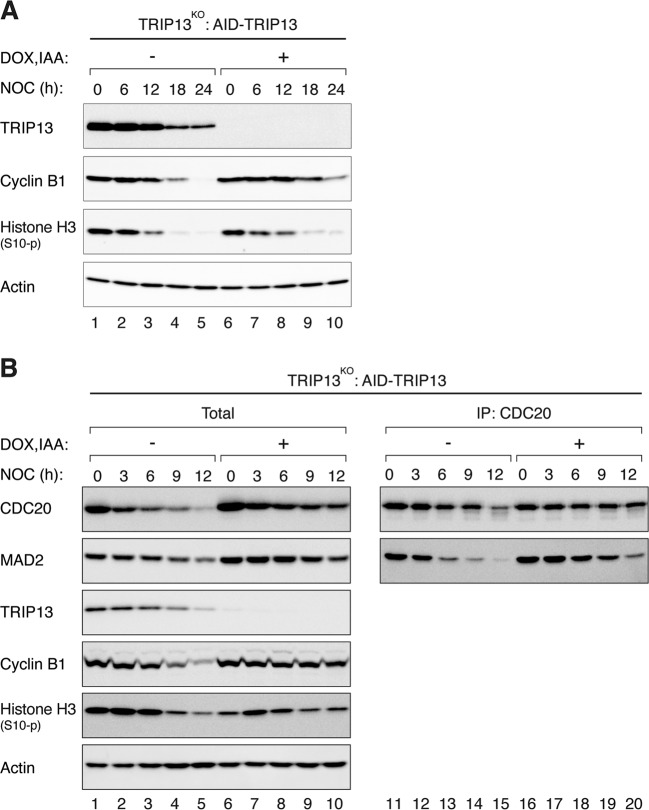
TRIP13 plays only a minor role in mitotic slippage. **a** TRIP13 only exerts a minor effect on mitotic slippage. TRIP13^KO^ cells expressing AID-TRIP13 were synchronized and treated with NOC as described in Fig. 1c. IAA and Dox were added at 6 h after addition of the second thymidine block. Lysates were prepared at the indicated time and analyzed with immunoblotting. **b** Reduction of MCC during mitotic arrest is only marginally affected by the absence of TRIP13. Cells were treated with NOC as described in **a** and harvested at the indicated time points. Lysates were prepared and subjected to immunoprecipitation (IP) using antiserum against CDC20 followed by immunoblotting analysis.

The above data suggested that p31^comet^ normally promotes mitotic slippage through a TRIP13-indepenent mechanism. To test the role of TRIP13 in mitotic slippage directly, a TRIP13^KO^ cell line expressing auxin-inducible degron (AID)-tagged TRIP13 was used in the mitotic slippage assays. Figure 5a shows that in the absence of AID-TRIP13 (by incubating with IAA and Dox), mitotic slippage occurred only marginally slower than in the presence of TRIP13. The decrease of cyclin B1, but not histone H3^Ser10^ phosphorylation, was slower in the absence of TRIP13. Likewise, although the reduction of MAD2–CDC20 complexes was slower in the absence of TRIP13 (Fig. 5b), it was less impressive than in p31^comet^-deficient cells (see Fig. 3c). Furthermore, no significant difference in the rate of mitotic slippage was detected using live-cell imaging analysis before and after TRIP13 degradation (Fig. S6A). Finally, the converse experiment of overexpressing TRIP13 also did not affect the rate of mitotic slippage (Fig. S6B).

Collectively, these data suggested that p31^comet^’s rate-determining function in mitotic slippage was mainly through putting a break on MAD2 activation. Inactivation of C-MAD2 by p31^comet^–TRIP13 mechanism plays a relatively minor role in the timing of mitotic slippage.

### Weakening of SAC during mitotic arrest involves reduction of MAD2 at the kinetochores

**Fig. 6:**
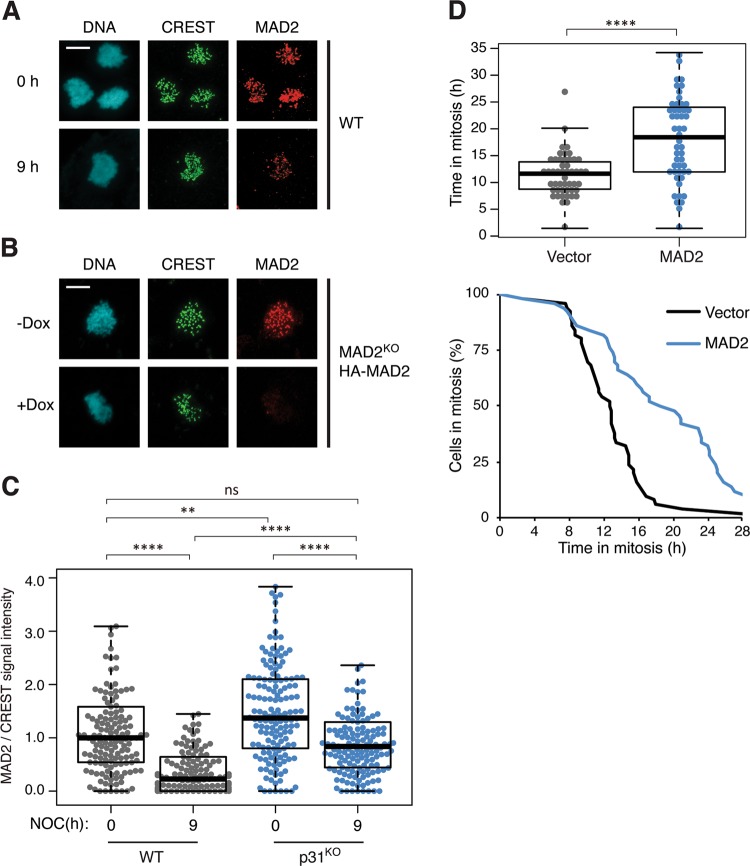
Weakening of SAC during mitotic arrest involves reduction of MAD2 at the kinetochores. **a** Reduction of kinetochore MAD2 during mitotic arrest. HeLa cells expressing BCL-2 were synchronized and treated with NOC as described in Fig. 1c. At *t* = 0 and 9 h after NOC treatment, mitotic cells were isolated, fixed, and analyzed using immunostaining for MAD2 and CREST. DNA was stained with Hoechst 33342. Examples of confocal microscopy images are shown. Scale bar: 10 µm. **b** Specificity of the MAD2 antibodies. MAD2^KO^ cells expressing HA-MAD2 were cultured in the absence or presence of Dox to turn on or off the HA-MAD2, respectively. The cells were first blocked in G_2_ using RO3306, before released for 1 h and incubated with NOC and MG132 for 2 h. Mitotic cells were isolated, fixed, and analyzed using immunostaining for MAD2 and CREST. DNA was stained with Hoechst 33342. Examples of confocal microscopy images are shown. Scale bar: 10 µm. **c** Kinetochore MAD2 is present at a higher level in p31^comet^-deficient cells than normal cells. The abundance of kinetochore MAD2 was analyzed in WT and p31^KO^ cells (both expressing FLAG-BCL-2) as described in **a**. The signal intensity of MAD2 at kinetochores was normalized with that of CREST (*n* = 150). Outliers that are higher than 1.5 times of the upper quartile and less than 1.5 times of the lower quartile are removed. **d** Overexpression of MAD2 delays mitotic slippage. HeLa cells expressing both BCL-2 and histone H2B-GFP were transiently transfected with either control vector or one expressing HA-MAD2 (a ECFP-expressing plasmid was co-transfected as a marker). Individual transfected cells were then tracked using live-cell imaging. The duration of mitotic arrest and the elapsed time between mitotic entry and mitotic cell death/slippage are shown (*n* = 50).

The above data show that the inactivation of SAC is a causal factor for mitotic slippage. Given that there is no evidence of an increase in SAC-inactivating mechanism during mitotic arrest—the abundance of p31^comet^ and TRIP13 remains unaltered (Fig. 1d)—we next investigated if the activation of SAC is compromised during mitotic arrest. The level of MAD2 at kinetochores was quantified using immunostaining and normalized using CREST signals. Significantly, we found that the abundance of kinetochore MAD2 was lower at late mitotic arrest compare with early mitotic arrest (Fig. 6a; quantified in Fig. 6c). Note that the total expression of MAD2 was not reduced over time (Fig. 1d). Addition of fresh NOC during the mitotic arrest did not affect the reduction of kinetochore MAD2 over time, indicating that the reduction of MAD2 was not merely due to drug inactivation (data not shown). These results suggest that a decrease in kinetochore MAD2 during mitotic arrest may be responsible for SAC inactivation and mitotic slippage.

To ensure that the immunostaining signals were indeed specific for MAD2, we used the same antibodies to analyze a conditional MAD2-deficient cell line. MAD2^KO^ cells expressing HA-MAD2 at a similar level as the endogenous MAD2 were generated (Fig. S7A). As the HA-MAD2 was under the control of a tetracycline-regulated promoter, it could be turned off in the presence of Dox. Immunostaining of mitotic cells lacking MAD2 (mitotic exit was prevented with a proteasome inhibitor) demonstrated the relatively high specificity of the MAD2 antibodies used here (Figs. 6b and S7B).

Given that mitotic slippage was significantly delayed in the absent of p31^comet^ (Fig. 3), we also analyzed the levels of kinetochore MAD2 in p31^comet^-deficient cells. Interestingly, kinetochore MAD2 signals also decreased over time in mitotic arrested p31^KO^ cells (Fig. 6c). Nevertheless, as the signals of kinetochore MAD2 during early mitotic arrest was already higher in p31^KO^ cells compare with WT cells, they were still significantly higher than that in WT cells at late mitosis (the signals at *t* = 9 h in p31^KO^ cells were comparable to that of *t* = 0 h in WT cells). Finally, consistent with the idea that a decrease in kinetochore MAD2 may be responsible for mitotic slippage, mitotic slippage was delayed by ectopic expression of HA-MAD2 (Fig. 6d).

Collectively, these results suggest that SAC is progressively weakened during mitotic arrest. Although the absence of p31^comet^ does not prevent the decrease of kinetochore MAD2, the increased level of kinetochore MAD2 at the onset of mitosis results in a delay in mitotic slippage.

### Feedback control to the SAC by APC/C-dependent activity during mitotic arrest

**Fig. 7:**
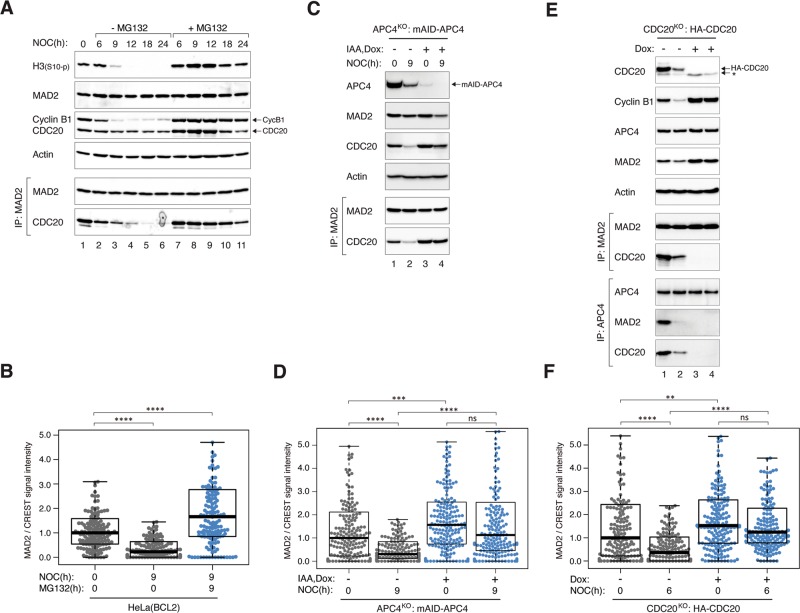
Weakening of SAC during mitotic arrest is dependent on APC/C and proteasome. **a** MG132 delays MCC inactivation and mitotic slippage. HeLa cells were synchronized and treated with NOC as described in Fig. 1c. The cells were incubated with either buffer or MG132. Lysates were prepared from cells harvested at the indicated time points and subjected to immunoprecipitation and immunoblotting. **b** Reduction of kinetochore MAD2 during mitotic arrest is proteasome-dependent. HeLa cells expressing BCL-2 were synchronized and treated with NOC either in the absence or presence of MG132 as described in Fig. 1c. At *t* = 0 and 9 h, mitotic cells were isolated, fixed, and analyzed by immunostaining. The signal intensity of MAD2 at kinetochores was normalized with that of CREST (*n* = 125). **c** APC/C activity is required for MCC inactivation. APC4^KO^ expressing mAID-APC4 cells were synchronized and treated with NOC as described in Fig. 1c with minor modifications: IAA and Dox were introduced (at 15 h after second thymidine release) to turn off mAID-APC4. Lysates were prepared at different time points and analyzed with immunoprecipitation and immunoblotting. **d** Reduction of kinetochore MAD2 during mitotic arrest is independent on APC/C. APC4^KO^ expressing mAID-APC4 cells were synchronized and treated with NOC as in **c**. At *t* = 0 and 9 h, mitotic cells were isolated, fixed, and analyzed using immunostaining. The signal intensity of MAD2 at kinetochores was normalized with that of CREST (*n* = 200). **e** Conditional depletion of CDC20. CDC20^KO^ cells expressing HA-CDC20 were synchronized as described in Fig. 1c with minor modifications: Dox was added to turn off the expression of HA-CDC20 (at 14 h after first thymidine release). Lysates were prepared from cells harvested at the indicated time points and subjected to immunoprecipitation (IP) using antiserum against MAD2 or APC4. The immunoprecipitates and total lysates were then analyzed using immunoblotting. The asterisk indicates the position of a cross-reactive band by the CDC20 antibodies (the band was absent in the MAD2 immunoprecipitates). **f** Reduction of kinetochore MAD2 during mitotic arrest is independent on CDC20. CDC20^KO^ cells expressing HA-CDC20 were synchronized were synchronized and treated with NOC as described in Fig. 1c with minor modifications: Dox were introduced (at 15 h after second thymidine release) to turn off HA-CDC20. At *t* = 0 and 6 h, mitotic cells were isolated, fixed, and analyzed using immunostaining. The signal intensity of MAD2 at kinetochores was normalized with that of CREST (*n* = 180).

The above imaging data indicated that mitotic slippage was dependent on proteasome activity (Figs. 1e and S1C, D). The delay in mitotic slippage in the presence of the proteasome inhibitor MG132 was confirmed by the sustained phosphorylation of histone H3^Ser10^ and stabilization of cyclin B1 (Fig. 7a). Moreover, the dissociation of MAD2–CDC20 complexes during mitotic arrest was delayed in the presence of MG132 (Fig. 7a). To determine if proteasome also affects the reduction of kinetochore MAD2 during mitotic arrest, we examined the staining of kinetochore MAD2 in the presence or absence of MG132. Significantly, the reduction of kinetochore MAD2 after prolonged mitotic arrest was abolished in the presence of MG132 (Fig. 7b).

To address if APC/C also contributed to the weakening of SAC during mitotic arrest, we made use of the APC4^KO^ cells expressing mAID-APC4 described above. While MAD2–CDC20 complexes were reduced over time in the presence of APC4, they were stabilized in the absence of APC4 (Fig. 7c), suggesting APC/C-dependency of MCC inactivation. In agreement with the targeting of CDC20 for degradation by APC/C during SAC activation [16], CDC20 was stabilized in APC4^KO^ cells (Fig. 7c). This may in part be responsible for the stabilization of MAD2–CDC20 complexes. Moreover, the reduction of the signals of kinetochore MAD2 over time was also compromised in APC4^KO^ cells (Fig. 7d).

A possible explanation is that destruction of CDC20 by APC/C during mitotic arrest was involved in the decrease of kinetochore MAD2. To shed light on this possibility, CDC20^KO^ cells expressing HA-CDC20 were blocked in mitosis using NOC and turning off the HA-CDC20 (Fig. 7e). As shown above, the abundance of MAD2–CDC20 complexes reduced during mitotic arrest in WT cells, which was also reflected in the reduction of MAD2 and CDC20 binding to APC/C (APC4 immunoprecipitates). As expected, the absence of MAD2–CDC20 complexes was accompanied with the loss of MAD2 binding to APC/C and stabilization of cyclin B1. In the absence of CDC20, MAD2 was still able to accumulate at kinetochores during mitotic block. Moreover, the decrease of MAD2 over time was abolished in the absence of CDC20 (Fig. 7f). These data provide evidence of a feedback mechanism involving APC/C-mediated decrease of kinetochore MAD2 during prolonged mitotic arrest.

## Discussion

**Fig. 8:**
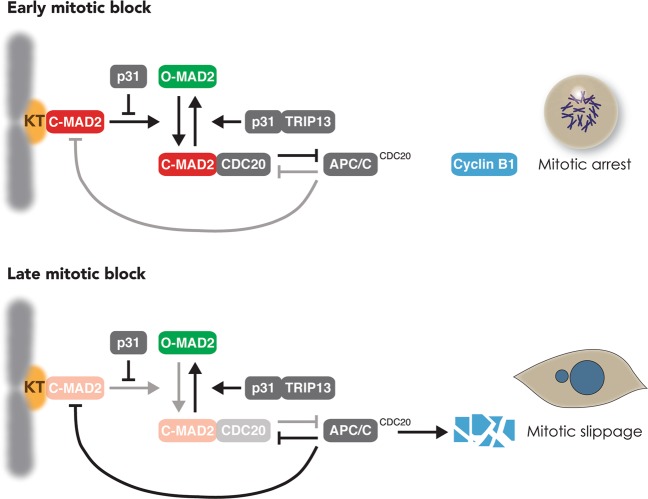
The proposed role of the weakening SAC in mitotic slippage. SAC is activated during early mitotic arrest: C-MAD2 at unattached kinetochores (KT) promotes the conversion of O- to C-MAD2, resulting in the formation of MCC, APC/C^CDC20^ inhibition, and cyclin B1 stabilization. This study provides evidence that the timing of mitotic slippage is controlled by the level of p31^comet^. This is achieved by the blocking MAD2 activation by p31^comet^, instead of the conversion of C- to O-MAD2 by p31^comet^–TRIP13. We also provide evidence that during late mitotic arrest, the level of MAD2 is reduced at unattached kinetochores. The progressive weakening of SAC during mitotic arrest enables APC/C^CDC20^ to gradually degrade cyclin B1, cumulating in mitotic slippage.

In this study, we provide evidence that gradual weakening of SAC is an integral mechanism of mitotic slippage (a model is shown in Fig. 8). During early mitotic arrest, MAD1–MAD2 complexes at unattached kinetochores promote the conversion of O-MAD2 into C-MAD2, initiating events including the coupling of MAD2 to CDC20, formation of MCC, and inhibition of APC/C^CDC20^. The ensuing stabilization of APC/C^CDC20^ substrates including cyclin B1 maintains the cell in a mitotic state. During early mitotic block, activation of SAC is able to overcome SAC-inactivating mechanisms including p31^comet^ and TRIP13. During prolonged mitotic arrest, however, MAD2 activation at unattached kinetochores is progressively weakened, resulting in a reduction of MCC. This may enable APC/C^CDC20^ to gradually degrade cyclin B1, eventually reducing it to a level insufficient for maintaining mitosis.

As cells trapped in mitosis can undergo either mitotic cell death or mitotic slippage [9], BCL-2-overexpressing cells were used in this study to switch the balance toward mitotic slippage (Fig. 1). An extended period of mitotic arrest before mitotic slippage has the advantage of providing a better temporal resolution for various analyses compared with cell lines that normally undergo rapid mitotic slippage. A caveat is that the assumption that mitotic cell death and mitotic slippage are two independent events is still debatable. For example, caspase activity has been reported to be required for the normal function of SAC [17–20]. Furthermore, the stability of BUBR1 during mitotic arrest has been linked to caspase-3 [21–23], thus providing a potential link between apoptosis and mitotic slippage. Nevertheless, another study using RPE-1 cells concluded that caspase activity is not required for mitotic slippage [24]. Another caveat is that due to technical difficulty in generating the cell lines, BCL-2 was not overexpressed in some of the conditional gene inactivation cell lines used in this study.

Conceptually, the progressive decrease in cyclin B1 during mitotic arrest does not necessarily involve the same mechanism for cyclin B1 degradation employed during normal mitotic exit. Nevertheless, in agreement with previous results [25–28], we showed that cyclin B1 degradation during mitotic slippage was dependent on APC/C^CDC20^ (Figs. 2 and S2). As the activation of APC/C^CDC20^ during mitotic slippage is not due to the attachment of spindles to kinetochores as in normal mitosis, a fundamentally different upstream mechanism is involved in the inactivation of SAC. It has been suggested that the slow degradation of cyclin B1 is simply due to incomplete inhibition of APC/C by SAC [10]. Our data do not support this hypothesis as depletion of p31^comet^ could inhibit APC/C-dependent degradation of cyclin B1 (Fig. 3).

In accordance with the role of SAC in maintaining the mitotic state, manipulation of the levels of SAC components can alter the kinetics of mitotic slippage. For example, deletion of MAD2 resulted in rapid mitotic slippage [29]. Conversely, overexpression of MAD2 delayed mitotic slippage (Fig. 6d). Likewise, overexpression of p31^comet^ evoked accelerated mitotic slippage similar as MAD2 KO (Fig. S5). In fact, mitotic slippage could be brought forward by p31^comet^ expressed at a level only marginally higher than the endogenous protein (Fig. S4B). Mechanistically, mitotic slippage induced by p31^comet^ was caused by blocking MAD2 activation instead of TRIP13-dependent MAD2 inactivation, as p31(QF) mutant was unable to induce mitotic slippage (Fig. S5). This is in agreement with the relatively minor influence of the expression of TRIP13 on the timing of mitotic slippage (Fig. 5). Conversely, removal of p31^comet^ with siRNA (Fig. 3a) or gene disruption (Fig. 3b) delayed mitotic slippage. The loss of p31^comet^ was associated with an increase in MAD2–CDC20 complexes during mitotic arrest (Fig. 3c). Furthermore, MAD2–CDC20 complexes reduced at a slower rate during mitotic arrest in p31^comet^-deficient cells compared with WT cells. Possibly due to the higher initial level of MAD2–CDC20 complexes, SAC took longer to be inactivated even when the upstream signaling at the kinetochores was turned off (Fig. 3d). These results indicate that although the expression of p31^comet^ remained stable during mitotic arrest, it is a sensitive regulator of the timing of mitotic slippage.

While it is clear that manipulating the SAC could affect the timing of mitotic slippage, an important question is whether changes in SAC during mitotic arrest are responsible for mitotic slippage. The abundance of both kinetochore MAD2 (Fig. 6c), MAD2–CDC20 complexes (Fig. 1d), and MAD2–CDC20 binding to APC/C (Fig. 7e) reduced during mitotic arrest and before the onset of mitotic slippage. Given that SAC-inactivating mechanisms including p31^comet^ and TRIP13 remained constant during mitotic arrest, the weakening of SAC was likely to be initiated by the loss of MAD2 activation at the kinetochores. The underlying mechanisms of why the abundance of kinetochore MAD2 reduced over time can only be speculated at the moment. Several mechanisms have been described for removing kinetochore MAD2 during normal mitotic exit. For example, MAD2 is stripped away from kinetochores in a dynein-dependent fashion [30]; microtubules binding to the NDC80 complex dissociate MPS1 from kinetochores [31, 32]. Nevertheless, as these pathways involve binding of microtubules to kinetochores, they are unlikely to be responsible for mitotic slippage in the presence of antimicrotubule chemicals.

The precise nature of MAD2 staining at the kinetochores also remains to be defined. Although it is generally accepted that the kinetochore MAD2 signals comprise of MAD2 binding to MAD1, whether other MAD2 populations such as MAD2–CDC20 are also present is unclear. Conversion of O-MAD2 to C-MAD2 at kinetochores is likely to be coupled directly to CDC20 binding [29]. The finding that MAD2 signals were still present at kinetochores in CDC20^KO^ cells (Fig. 7f) argues against the signals that are normally contributed by MAD2–CDC20.

As the decrease in kinetochore MAD2 during mitotic arrest is dependent on the proteasome (Fig. 7b) and APC/C (Fig. 7d), one possibility is that similar to CDC20 [16], MAD2 itself is targeted to degradation by APC/C. This is unlikely to be a key mechanism because the expression of MAD2 was not affected by either APC4 KO (Fig. 7c) or after proteasome inhibition (Fig. 7a). It is more likely that progressive degradation of other kinetochore SAC components such as MPS1 [33] is responsible for the loss of MAD2 during mitotic arrest.

## Materials and methods

### DNA constructs

FLAG-3C-CDC20 in pUHD-P3 [34] and HA-MAD2 in pUHD-P2 [35] were generated as previously described. BCL-2 in pCMV-SPORT6 (Image ID:4511027) was obtained from Source Bioscience (Nottingham, UK). A FLAG-BCL-2 construct was generated by first amplifying BCL-2 cDNA with PCR using the primers 5′-AACCATGGCGCACGCTGGGAGAA-3′ and 5′-TGGAATTCTCACTTGTGGCCCAGATA-3′; the PCR product was cut with NcoI–EcoRI and ligated into pUHD-P3 [36]; the XhoI–EcoRI fragment of this construct was ligated into pUHD-P3T(PUR) [36] to obtain FLAG-BCL-2 in pUHD-P3T(PUR). Histone H2B-GFP construct was a gift from Tim Hunt (Cancer Research UK, UK). ECFP-expressing plasmid was a gift from Donald Chang (HKUST, Hong Kong). Constructs for expressing FLAG- or HA-p31^comet^ (WT and mutants) and TRIP13(WB) are as previously described [4]. APC4 CRISPR–Cas9 targeting an exon sequence (5′-TTAAGCTCTTGGGAGACGTC-3′) was prepared by ligating the annealed product of 5′-CACCGTTAAGCTCTTGGGAGACGTC-3′ and 5′-AAACGACGTCTCCCAAGAGCTTAAC-3′ to BbsI-cut pX330 (obtained from Addgene, Cambridge, MA, USA). To generate CRISPR-resistant APC4 expression construct, APC4 cDNA containing silent mutations were prepared by double PCR using the following pairs of primers: 5′-CCGAATTCATGTTGCGTTTTCCGACC-3′ and 5′-GACATCGCCTAAGAGCTTAAT-3′; 5′-GCTCTTAGGCGATGTCAGGCTT-3′ and 5′-ATGGATCCCACAATAATGGCAAGCTAGA-3′. The PCR product was digested with EcoRI and BamHI, and then ligated into EcoRI- and BamHI-cut pRevTRE-mAID. The pRevTRE-mAID was generated as described for pRevTRE-AID [37] except that mAID (a gift from Helfrid Hochegger, University of Sussex, UK) was used instead of AID. CDC20 CRISPR–Cas9 targeting a intron-exon boundary sequence (5′-CAGTCTGTTCTGATAACCTG-3′) was prepared by ligating the annealed product of 5′-CACCGCAGTCTGTTCTGATAACCTG-3′ and 5′-AAACCAGGTTATCAGAACAGACTGC-3′ to BbsI-cut pX330. HA-CDC20 was generated by ligation of the CDC20 cDNA obtained from partial digestion of FLAG-3C-CDC20 [34] with NcoI and XbaI into NcoI- and XbaI-cut pUHD-P2 [38]. A puromycin-resistant cassette was inserted into the BamHI site to create HA-CDC20 in pUHD-P2/PUR.

### Cell culture

The HeLa used in this study was a clone expressing the tTA tetracycline transactivator [38]. H1299 cells were obtained from American Type Culture Collection (Manassas, VA, USA). Cells were propagated in Dulbecco’s modified Eagle’s medium supplemented with 10% (v/v) calf serum (for HeLa) or fetal bovine serum (for H1299) and 50 U/ml of penicillin streptomycin (Life Technologies, Carlsbad, CA, USA). Cells were cultured in humidified incubators at 37 °C with 5% CO_2_. Cells were treated with the following reagents at the indicated final concentration: Dox (2 µg/ml), IAA (50 µg/ml), MG132 (10 µM), nocodazole (100 ng/ml) (Sigma-Aldrich, St. Louis, MO, USA), AZ 3146 (1 µM) (Selleck Chemicals, Houston, TX, USA), and Z-VAD-FMK (pan-caspase inhibitor) (10 µM; Enzo Life Sciences, Farmingdale, NY, USA). Transfection was carried out using a calcium phosphate precipitation method [39]. Synchronization with a double-thymidine procedure was as previously described [40].

### Stable cell lines

HeLa cells lacking p31^comet^ (with or without FLAG-p31^comet^ or p31^comet^ mutants) were generated as previously described [4]. Cells overexpressing BCL-2 were generated by transfecting HeLa or p31^KO^ cells with FLAG-BCL-2 in pUHD-P3T(PUR). The transfected cells were enriched by growing in puromycin-containing medium (1 µg/ml) for 5 days followed with medium without puromycin for 5 days. The cells were then seeded onto 96-well plates with limiting dilution to obtain single cell-derived colonies. The colonies were then analyzed with immunoblotting to confirm the presence of FLAG-BCL-2. To generate cells stably expressing histone H2B-GFP, different cell lines were transfected with histone H2B-GFP construct and grown in the presence of blasticidin (5 µg/ml). After 2 weeks, individual colonies were isolated and propagated in the absence of blasticidin. FUCCI-expressing cells were as described [41]. MAD2^KO^ expressing HA-MAD2 and TRIP13^KO^ expressing AID-TRIP13 were as described previously [29]. HA-CDC20 in CDC20^KO^ cells were generated by transfecting HeLa cells with CDC20 CRISPR–Cas9 in pX330 and HA-CDC20 in pUHD-P2/PUR plasmids. The transfected cells were selected by growing in puromycin-containing medium for 10 days followed by drug-free medium for 5 days. The CDC20 CRISPR–Cas9 was transfected a second time together with a plasmid expressing blasticidin-resistant gene (a gift from Tim Hunt) before selected by growing in blasticidin-containing medium for 2 days. The cells were then seeded onto 96-well plates with limiting dilution to obtain single cell-derived colonies. APC4^KO^ cells expressing mAID-APC4 were generated by first infecting HeLa cells with retroviruses expressing mAID-APC4 (possessing silent mutations to the CRISPR) and grown in medium containing hygromycin B (250 µg/ml) for 2 weeks. The cells were then co-transfected with APC4 CRISPR–Cas9 in pX330 and a plasmid expressing blasticidin-resistant gene. After enriching the transfected cells with blasticidin selection for 36 h (5 µg/ml), the cells were infected with retroviruses expressing TIR1-myc. The cells were selected with puromycin and single cell-derived colonies were obtained by limiting dilution in 96-well plates.

### RNA interference

Stealth siRNA targeting CDH1 (UCAACCUCUUCACCAGGAUCCGGUA), CDC20 (GCACCAGUGAUCGACACAUUCGCAU), p31^comet^ (GGAGUGGUAUGAGAAGUCCGAAGAA), and control siRNA were manufactured by Life Technologies. Cells were transfected with siRNAs (15 nM) using Lipofectamine^TM^ RNAiMAX (Life Technologies).

### Antibodies and immunological methods

Immunoblotting was performed as previously described [42], except that a ChemiDoc Touch imaging system (Bio-Rad, Hercules, CA, USA) was used to detect the signals. The following antibodies were obtained from the indicated sources and used at the indicated concentrations: monoclonal antibodies against beta-actin (Sigma-Aldrich; 0.2 µg/ml), FLAG (Sigma-Aldrich; 1 µg/ml), BUB3 (BD Biosciences, Franklin Lakes, NJ, USA; 0.25 µg/ml), CDC27 (BD Biosciences; 0.125 µg/ml), cleaved PARP1(Asp214) (BD Biosciences; 0.2 µg/ml), CDH1 (Thermo Scientific; 1 µg/ml), CDC20 (Santa Cruz Biotechnology, Santa Cruz, CA, USA; 0.2 µg/ml), MAD1 (Santa Cruz Biotechnology; 0.2 µg/ml),TRIP13 (Santa Cruz Biotechnology; 0.2 µg/ml), APC4 (Abcam, Cambridge, UK; 0.1 µg/ml), polyclonal antibodies against phosphor-histone H3^Ser10^ (Santa Cruz Biotechnology; 0.1 µg/ml), BUBR1 (Bethyl Laboratories, Montgomery, TX, USA; 0.2 µg/ml), MAD2 [43], and p31^comet^ [44]. Antibodies against cyclin B1 (1 µg/ml) were gifts from Julian Gannon (Cancer Research UK). Immunoprecipitation of MAD2 or CDC20 was performed as previously described [43]. Antibody against APC4 for immunoprecipitation was prepared by immunizing rabbits with an APC4 peptide (CIVIKVEKLDPELDS) (GenScript, Piscataway, NJ, USA).

### Immunostaining

Cells were collected by centrifugation and resuspended in medium to a concentration of 300 cells/μl. The cell suspension (400 μl) was then applied to a CellSpin cytocentrifuge (Tharmac, Waldsolms, Germany) and centrifuged at 500 rpm for 5 min. The coverslips were then fixed with 2% formaldehyde, 0.1% Triton-X in PBS at 25 °C for 10 min before washed with PBS (all washes were three times, 5 min each). The samples were blocked with blocking buffer (1% BSA, 0.1% Triton-X in PBS) for 30 min, washed with PBST (0.1% Triton-X in PBS), and incubated with primary antibodies diluted in blocking buffer at 25 °C for 2 h. The samples were then washed with PBST (three times), incubated with autoantibody against human nuclear ANA-centromere CREST (Fitzgerald Industries, Acton, MA, USA; 1:500, 25 °C for 2 h), before washed again with PBST. The samples were incubated with Alexa-Flour-568 goat anti-rabbit IgG and Alexa-Flour-647 goat anti-human IgG (Life Technologies) at 25 °C for 2 h, washed with PBST, and stained with Hoechst 33342 (Life Technologies) at 1:10,000 dilution in PBST for 10 min. After washing with PBST, the coverslips were mounted with 2% N-propyl-gallate (Sigma-Aldrich) in glycerol. Images were taken with a TE2000E-PFS microscope (Nikon, Tokyo, Japan) equipped with a SPOT BOOST EMCCD camera (SPOT Imaging Solutions, Sterling Heights, MI, USA) or with a Leica Sp8 Confocal Microscope (Leica Microsystem, Wetzlar, Germany). ImageJ software (National Institutes of Health, Bethesda, MD, USA) was used to process the images. Signals were quantified using CellProfiler (Version 3.1.5) [45]. The integrated pixel intensities of CREST and MAD2 foci (within CREST foci) were measured.

### Live-cell imaging

Cells were seeded onto 24- or 96-well cell culture plates, and the plates were placed onto a TE2000E-PFS microscope (Nikon, Tokyo, Japan) equipped with an Andor Zyla sCMOS camera (Oxford Instruments, UK) and a Chamlide TC temperature, humidity, and CO_2_ control chamber (Live Cell Instrument, Seoul, South Korea). Images were captured every 5 min for 24 h or every 10 min for 48 h. Data acquisition was carried out with Metamorph 7.8.6 software (Molecular Devices, Sunnyvale, CA, USA) and analysis was performed using ImageJ software (National Institutes of Health, Bethesda, MD, USA). For histone H2B-GFP-expressing cells, the duration of mitosis was estimated from the time of DNA condensation to decondensation. For FUCCI-expressing cells, the duration of mitosis was estimated from the time of the APC/C reporter dispersing from the nucleus (due to nuclear envelope breakdown) to its destruction.

### Flow cytometry

Flow cytometry analysis after propidium iodide staining was performed as previously described [46].

### Statistical analysis

Box-and-whisker plots (center lines show the medians; box limits indicate the interquartile range; whiskers extend to the most extreme data points that were no more than 1.5 times the interquartile range from the 25th and 75th percentiles) were generated using RStudio (version 1.1.456; Boston, MA, USA). For bee swarm plots, libraries ‘beeswam’ and ‘plyr’ were obtained from CRAN (outliers were removed in the plots; data points were corralled within certain range). Mann–Whitney–Wilcoxon test was used to calculate statistical significance (*p < 0.05; **p < 0.01; ***p < 0.001; ****p < 0.0001; ns *p* > 0.05).

## Supplementary information

Supplemental Figure Legends

Figure S1. Inhibition of apoptosis promotes proteasome-dependent mitotic slippage

Figure S2. CDC20 but not CDH1 is required for mitotic slippage

Figure S3. Mitotic slippage is delayed after knockdown of p31comet

Figure S4. p31comet controls the rate of mitotic slippage

Figure S5. Induction of mitotic slippage by p31comet does not require binding to TRIP13

Figure S6. Depletion or overexpression of TRIP13 does not affect mitotic slippage

Figure S7. The specificity of MAD2 antibodies
