# Supplementary material for: Mitotic slippage is determined by p31comet and the weakening of the spindle-assembly checkpoint
Source: Oncogene. 2020 Feb 6;39(13):2819–34. doi: 10.1038/s41388-020-1187-6 (PMC7098889; doi:10.1038/s41388-020-1187-6)
Supplement: Supplementary file 1 — Supplemental Figure Legends [file 41388_2020_1187_MOESM1_ESM.pdf]

SUPPLEMENTAL FIGURE LEGENDS

Figure S1. Inhibition of apoptosis promotes proteasome-dependent mitotic slippage.

(A) Inhibition of mitotic cell death. HeLa cells expressing FLAG-BCL-2 under the control of doxycycline (Dox) were cultured in the absence or presence of Dox to turn on or off the FLAG-BCL-2, respectively. The cells were then incubated with NOC and harvested at the indicated time points. Immunoblotting was used to analyze FLAG-BCL-2, apoptosis (cleaved PARP1), and phosphor-histone $H3^{Ser10}$ (note that histone $H3^{Ser10}$ phosphorylation also increased in apoptotic cells). Equal loading of lysates was confirmed by immunoblotting for actin.

(B) Inhibition of apoptosis promotes mitotic slippage and rereplication. HeLa (with or without stable expression of BCL-2) were incubated with NOC. At the indicated time points, the cells were harvested and analyzed with flow cytometry. The positions of 2N (G_1), 4N (G_2/M), and 8N (rereplicated) DNA contents are indicated. Note the extensive apoptosis (sub-G_1 population) in wild type HeLa cells.

(C) Delay of Mitotic slippage after inhibition of the proteasome. HeLa cells expressing both BCL-2 and histone H2B-GFP were synchronized and incubated with NOC as described in Fig 1C. The cells were incubated with either buffer or MG132. Individual cells were then tracked using live-cell imaging for 24 h. Key: interphase (grey); mitosis (red); interphase after mitotic slippage (green); truncated bars (cell death). For Fig 1E.

(D) Degradation of APC/C substrates during prolonged mitotic arrest is delayed with MG132. HeLa cells expressing both BCL-2 and the FUCCI cell cycle reporter system were synchronized and incubated with NOC as described in Fig 1C. The cells were incubated with either buffer or MG132. Individual cells were then tracked using live-cell imaging. The signal intensity

of the APC/C reporter (consists of a fragment of Geminin labelled with mVenus) in individual cells was quantified (normalized to the intensity at the time of mitotic entry; the period from mitotic entry to mitotic exit is shown).

Figure S2. CDC20 but not CDH1 is required for mitotic slippage.

(A) Downregulation of CDC20 delays mitotic slippage. HeLa cells expressing BCL-2 were transfected with siRNA against CDC20 and/or CDH1. The cells were treated with NOC as described in Fig 1C (transfection was performed at the first thymidine release) before analyzed using live-cell imaging. Key: interphase (grey); mitosis (red); interphase after mitotic slippage (green); truncated bars (cell death). The downregulation of CDC20 and/or CDH1 was confirmed using immunoblotting. For Fig 2B.

(B) Downregulation of CDC20 delays mitotic slippage in H1299 cells.H1299 cells expressing histone H2B-GFP were transfected with siRNA against CDC20 and/or CDH1. The cells were treated with NOC as described in Fig 1C in the presence of a caspase inhibitor before analyzed using live-cell imaging. Key: interphase (grey); mitosis (red); interphase after mitotic slippage (green); truncated bars (cell death). The duration of mitotic arrest and the elapsed time between mitotic entry and mitotic cell death/slippage are shown. The downregulation of CDC20 and/or CDH1 was confirmed using immunoblotting.

(C) Overexpression of CDC20 promotes mitotic slippage. Cells were transfected with either vector or a plasmid expressing FLAG-CDC20. A plasmid expressing ECFP was co-transfected. After 40 h, the cells were treated with NOC and analyzed using live-cell imaging. Key: interphase (grey); mitosis (red); interphase after mitotic slippage (green); truncated bars (cell death). The expression of FLAG-CDC20 (F-CDC20) was confirmed using immunoblotting. For Fig 2D.

Figure S3. Mitotic slippage is delayed after knockdown of p31^{comet}.

(A) Knockdown of p31^{comet}. HeLa (expressing BCL-2) and H1299 cells were transfected with either control or siRNA against p31^{comet} (sip31). After 48 h, the cells were harvested and analyzed with immunoblotting.

(B) HeLa cells expressing BCL-2 were transfected with either control or sip31. The cells were synchronized and incubated with NOC as described in Fig 1C. Individual cells were then tracked using live-cell imaging for 48 h. Key: interphase (grey); mitosis (red); interphase after mitotic slippage (green); truncated bars (cell death). Note that the control is the same as in Fig S2A. For Fig 3A.

(C) Mitotic slippage is delayed after knockdown of p31^{comet} in H1299 cells. H1299 cells expressing histone H2B-GFP were transfected with either control or sip31. After 48 h, the cells were treated with NOC and a caspase inhibitor before analyzed using live-cell imaging. Key: interphase (grey); mitosis (red); interphase after mitotic slippage (green); truncated bars (cell death). The duration of mitotic arrest and the elapsed time between mitotic entry and mitotic cell death/slippage are shown. Confirmation of the knockdown of p31^{comet} is shown in Fig S3A.

Figure S4. p31^{comet} controls the rate of mitotic slippage.

(A) Mitotic slippage is delayed in p31^{comet}-deficient cells. HeLa and p31^{comet}-deficient cells (p31^{KO}) (both stably expressing BCL-2) were incubated with NOC before analyzed using live-cell imaging for 48 h. Key: interphase (grey); mitosis (red); interphase after mitotic slippage (green); truncated bars (cell death). For Fig 3B.

(B) Reintroduction of p31^{comet} reverses the delay of mitotic slippage in p31^{comet}-deficient cells. The indicated cell lines were synchronized and

incubated with NOC (a pan-caspase inhibitor was applied at the same time) as described in Fig 1C. Individual cells were then tracked using live-cell imaging for 48 h. Key: interphase (grey); mitosis (red); interphase after mitotic slippage (green); truncated bars (cell death). The duration of mitotic arrest and the elapsed time between mitotic entry and mitotic cell death/slippage are shown. Lysates from the different cell lines were analyzed with immunoblotting.

Figure S5. Induction of mitotic slippage by p31^{comet} does not require binding to TRIP13.

(A) p31(QF) does not bind MAD2. FLAG (F)-tagged p31 or p31(QF) was transiently transfected into HeLa cells. Lysates were immunoprecipitated with anti-FLAG antibodies and analyzed with immunoblotting.

(B) p31(PK) does not bind TRIP13. HA-tagged p31 or p31(PK) was co-transfected with FLAG-TRIP13 into HeLa cells. A Walker B mutant (WB) of TRIP13 (which probably acts as a substrate trap for p31^{comet}–MAD2) was used. Lysates were immunoprecipitated with anti-FLAG antibodies and analyzed with immunoblotting.

(C) HeLa cells were transiently transfected with constructs expressing HA-tagged p31^{comet}, p31(QF), p31(PK), and p31(QF/PK) mutants (a ECFP-expressing construct was co-transfected as a marker). NOC was added at 40 h after transfection, Individual ECFP-positive cells were then tracked using live-cell imaging for 48 h. Key: interphase (grey); mitosis (red); interphase after mitotic slippage (green); truncated bars (cell death). The duration of mitotic arrest and the elapsed time between mitotic entry and mitotic cell death/slippage are shown. Immunoblotting analysis showing the expression of the different HA-p31^{comet} constructs.

Figure S6. Depletion or overexpression of TRIP13 does not affect mitotic slippage.

(A) Depletion of TRIP13 did not affect the rate of mitotic slippage. $TRIP13^{KO}$ cells expressing AID-TRIP13 were synchronized and incubated with NOC as described in Fig 1C before analyzed using live-cell imaging. IAA and Dox were applied at 6 h after addition of second thymidine block. The duration of mitotic arrest and the elapsed time between mitotic entry and mitotic cell death/slippage are shown (*n*=50).

(B) Overexpression of TRIP13 did not affect the rate of mitotic slippage. HeLa cells expressing both BCL-2 and histone H2B-GFP were transfected with either control vector or a plasmid expressing FLAG-TRIP13. A plasmid expressing ECFP was co-transfected. After 40 h, the cells were treated with NOC and analyzed using live-cell imaging. The duration of mitotic arrest and the elapsed time between mitotic entry and mitotic cell death/slippage are shown (*n*=33).

Figure S7. The specificity of MAD2 antibodies.

(A) Conditional depletion of MAD2. $MAD2^{KO}$ cells expressing HA-MAD2 were cultured in the absence or presence of Dox for 12 h to turn on or off the HA-MAD2, respectively. The cells were incubated with either buffer or NOC and MG132 for 12 h to trap cells in mitosis. Lysates were prepared and analyzed with immunoblotting.

(B) Specificity of the MAD2 antibodies. $MAD2^{KO}$ cells expressing HA-MAD2 were cultured in the absence or presence of Dox for 24 h to turn on or off the HA-MAD2, respectively. The cells were incubated with NOC and MG132 for 2 h. Mitotic cells were isolated, fixed, and analyzed using immunostaining using anti-MAD2 and anti-CREST antibodies. The signal intensity of MAD2 at kinetochores was normalized with that of CREST. For Fig 6B.
