# Supplementary figures and images for: Mitotic slippage is determined by p31comet and the weakening of the spindle-assembly checkpoint
Source: Oncogene. 2020 Feb 6;39(13):2819–34. doi: 10.1038/s41388-020-1187-6 (PMC7098889; doi:10.1038/s41388-020-1187-6)

**A**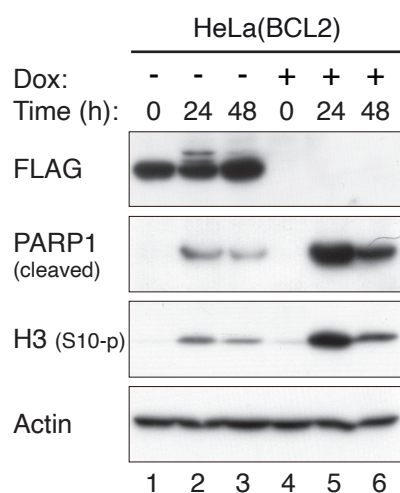**B**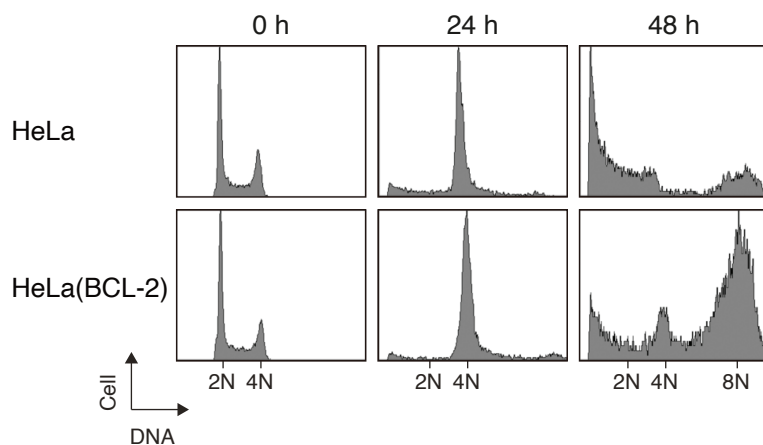**C**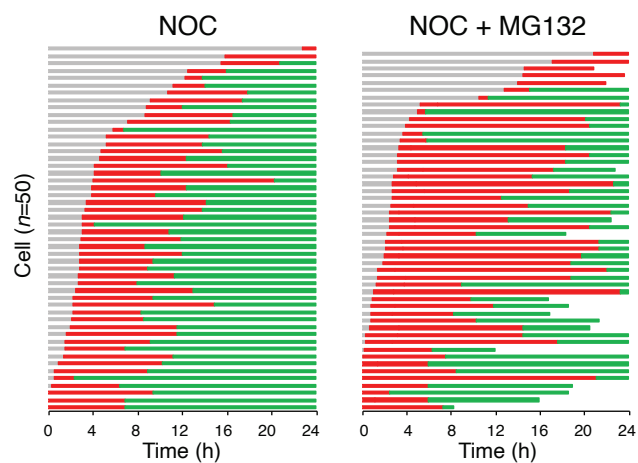**D**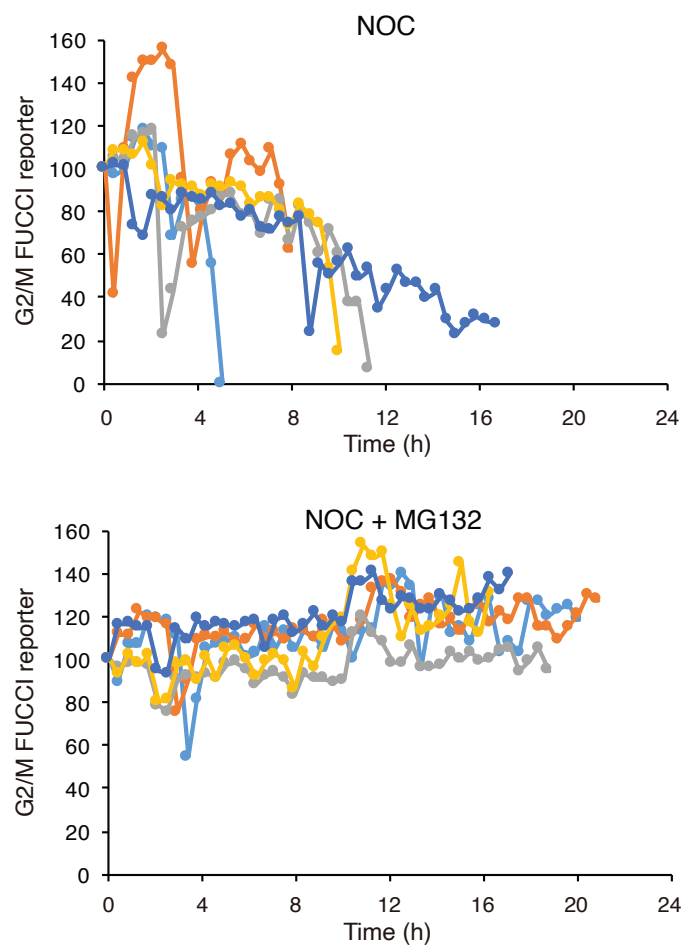

Supplement: Supplementary file 2 — Figure S1. Inhibition of apoptosis promotes proteasome-dependent mitotic slippage [file 41388_2020_1187_MOESM2_ESM.pdf]

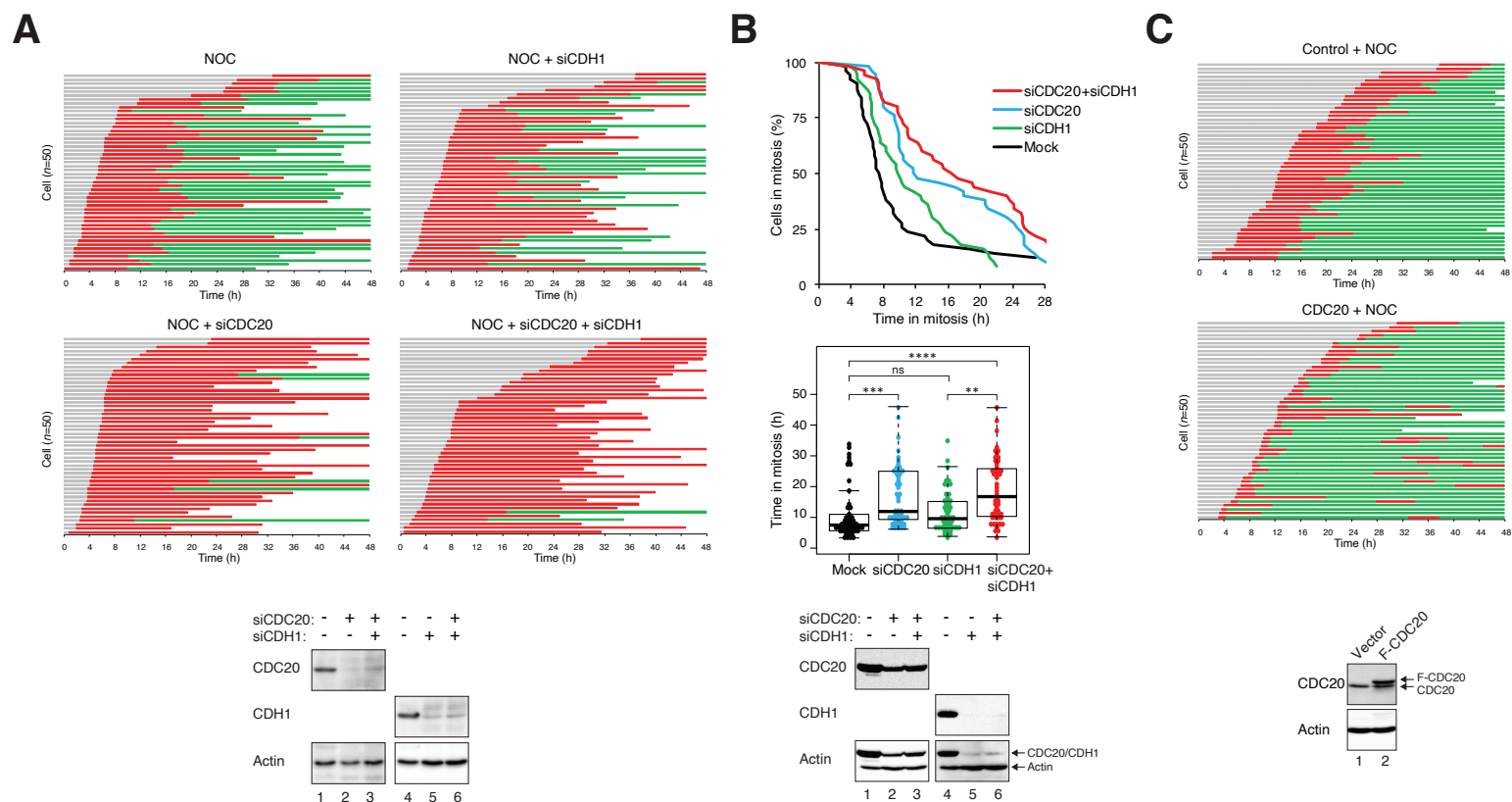

Supplemental Figure S2

Supplement: Supplementary file 3 — Figure S2. CDC20 but not CDH1 is required for mitotic slippage [file 41388_2020_1187_MOESM3_ESM.pdf]

**A**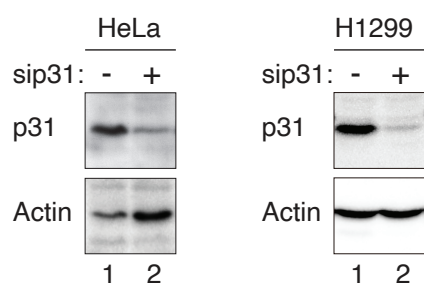**B**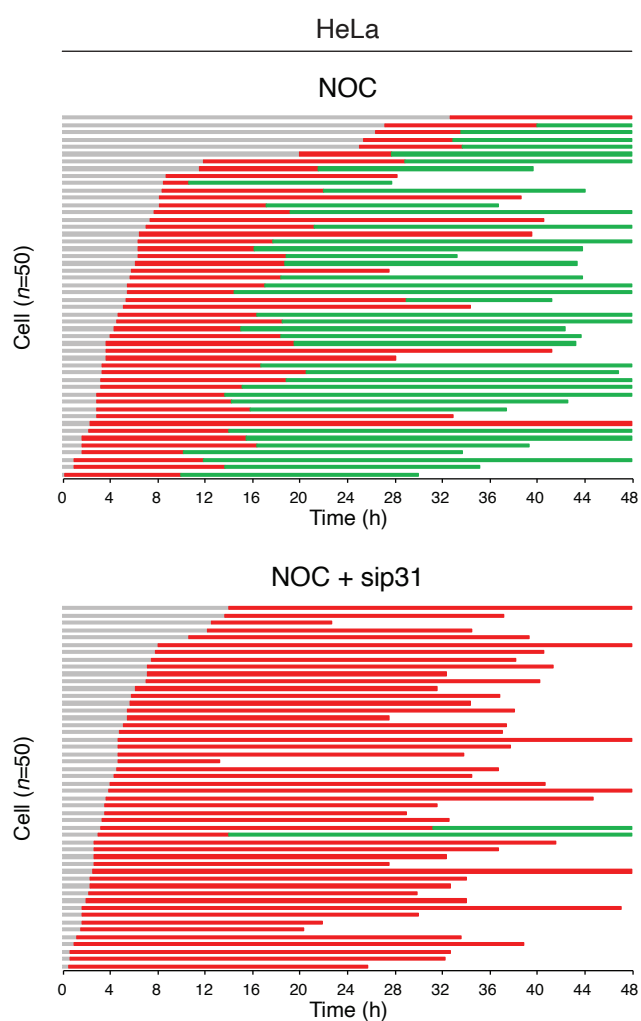**C**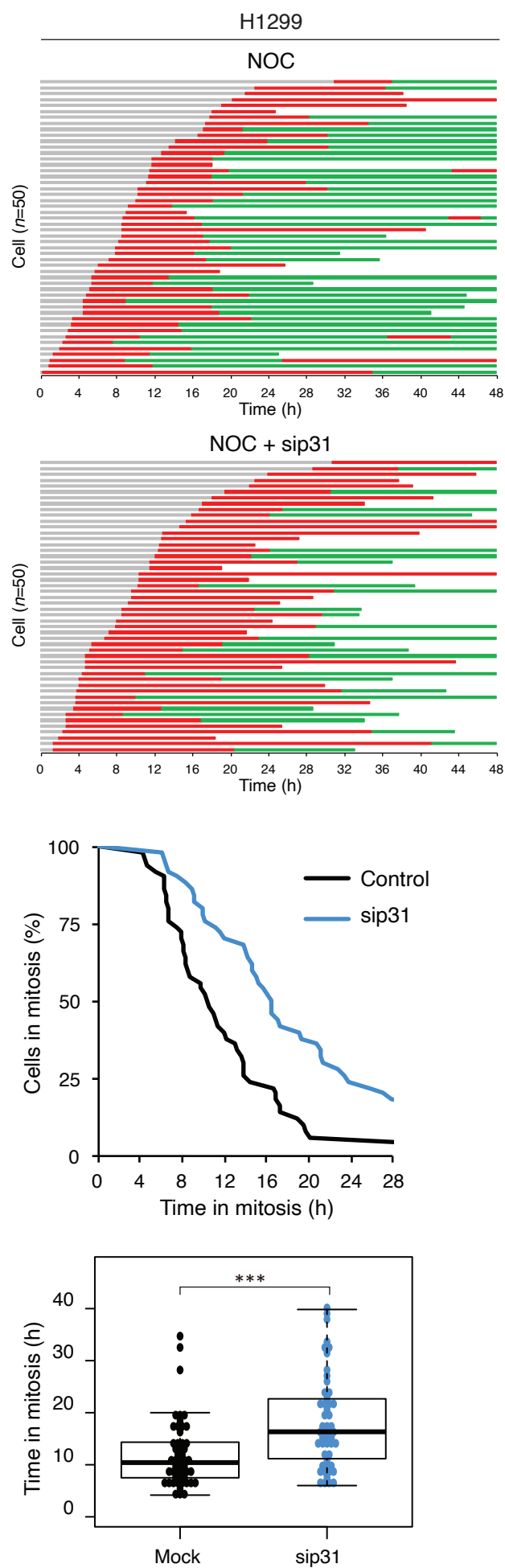

Supplemental Figure S3

Supplement: Supplementary file 4 — Figure S3. Mitotic slippage is delayed after knockdown of p31comet [file 41388_2020_1187_MOESM4_ESM.pdf]

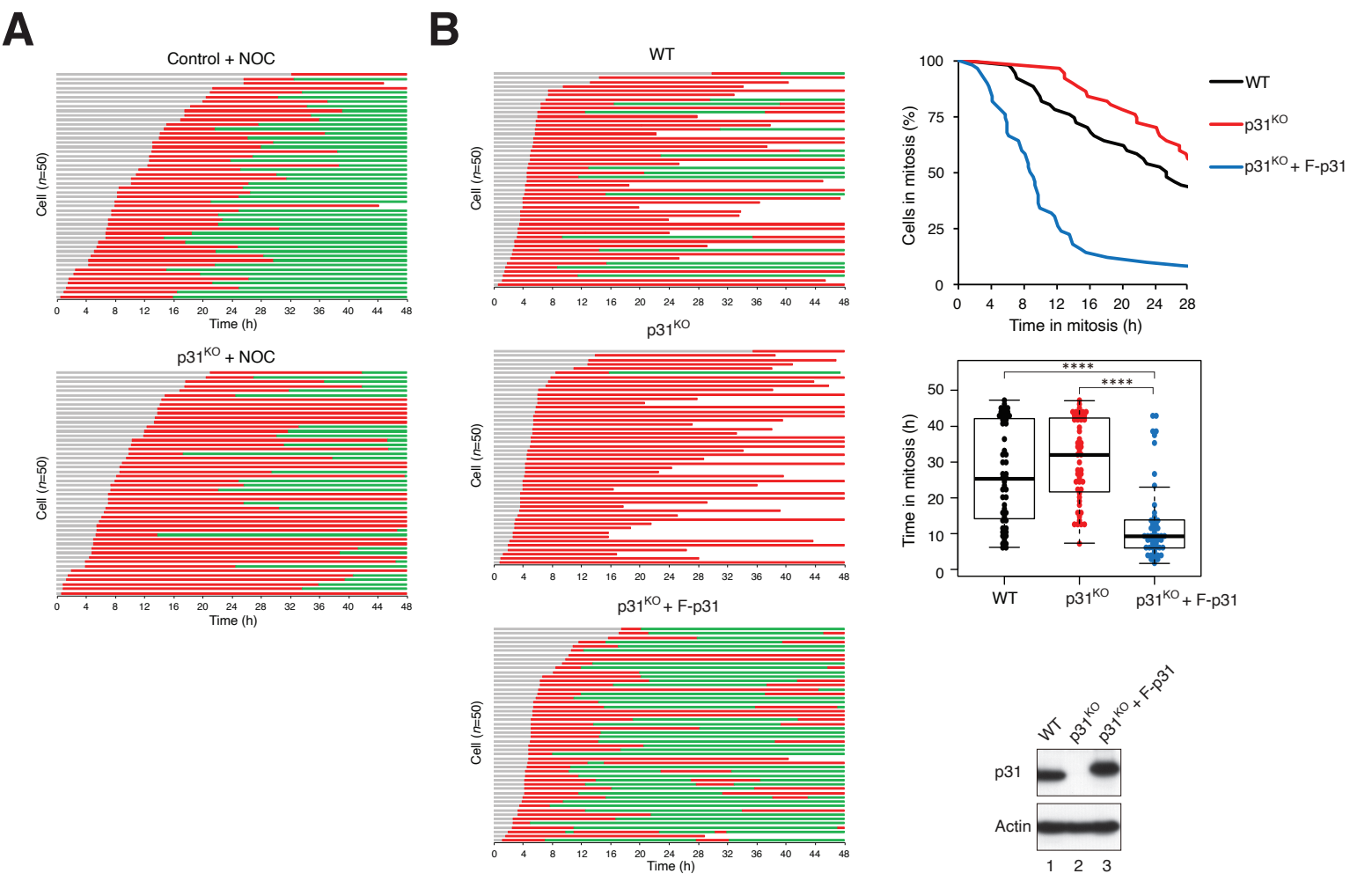

Supplemental Figure S4

Supplement: Supplementary file 5 — Figure S4. p31comet controls the rate of mitotic slippage [file 41388_2020_1187_MOESM5_ESM.pdf]

**A**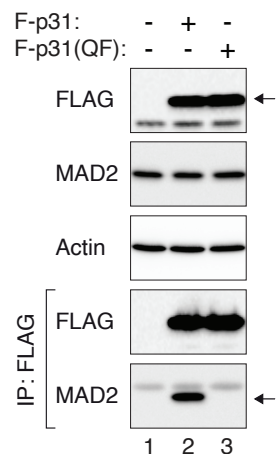**B**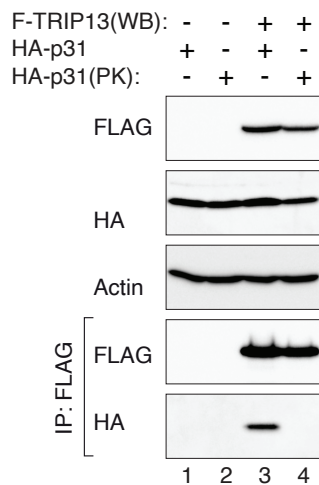**C**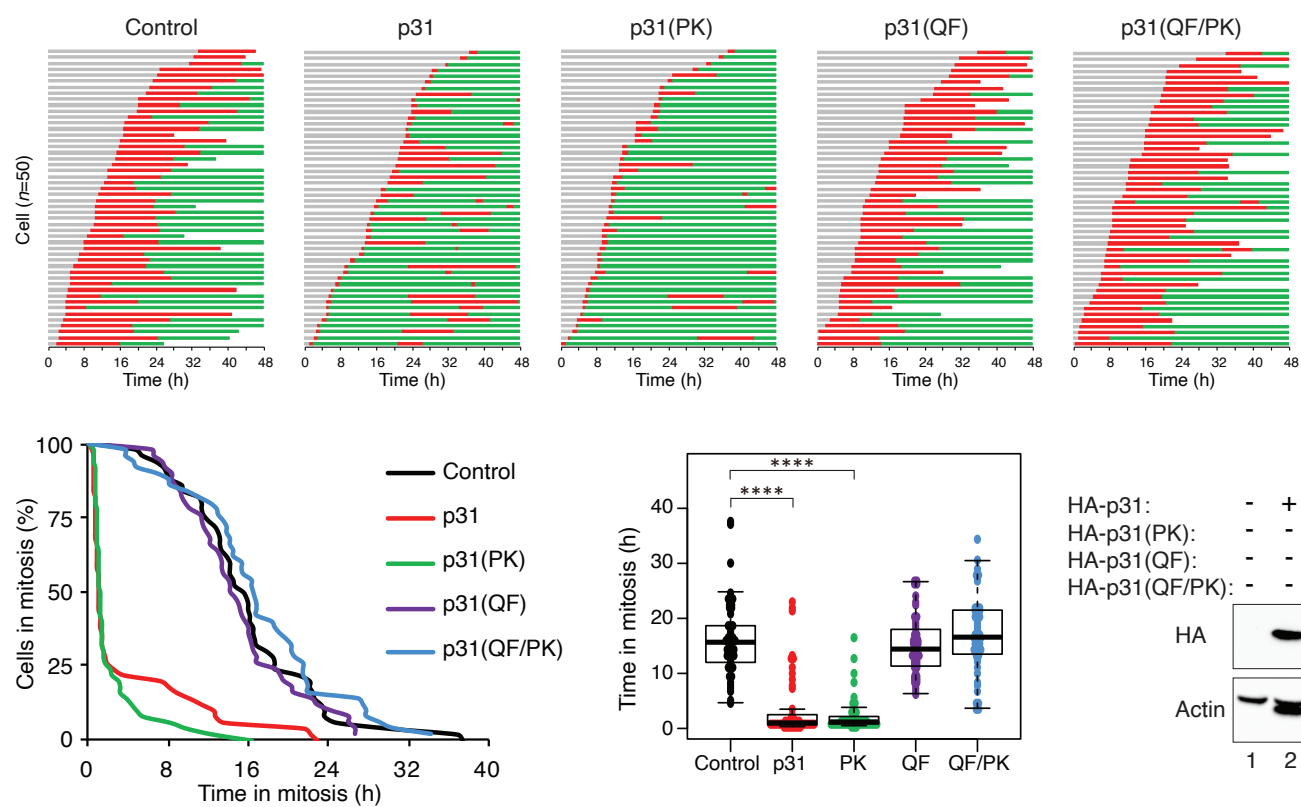

Supplemental Figure S5

Supplement: Supplementary file 6 — Figure S5. Induction of mitotic slippage by p31comet does not require binding to TRIP13 [file 41388_2020_1187_MOESM6_ESM.pdf]

**A**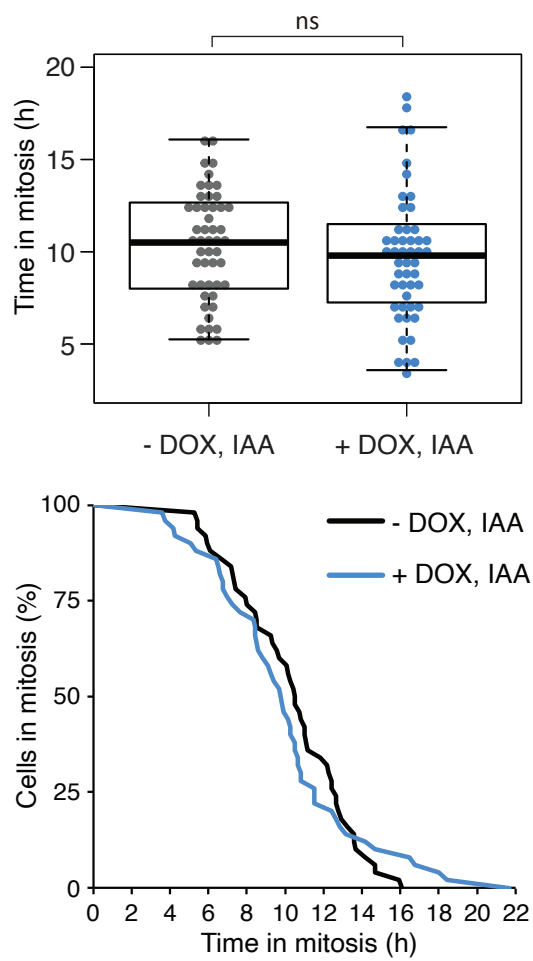**B**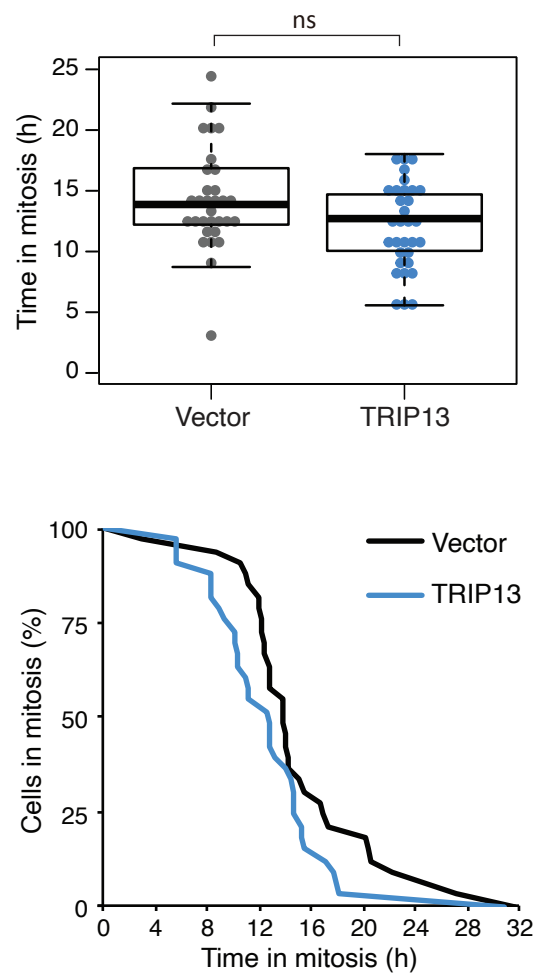

Supplement: Supplementary file 7 — Figure S6. Depletion or overexpression of TRIP13 does not affect mitotic slippage [file 41388_2020_1187_MOESM7_ESM.pdf]

**A**

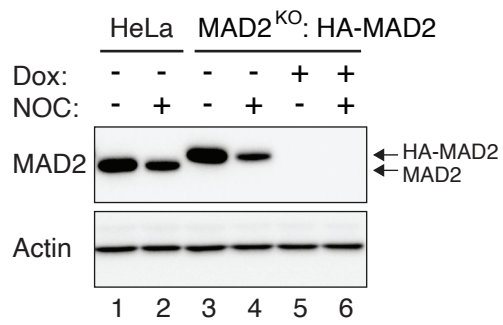

**B**

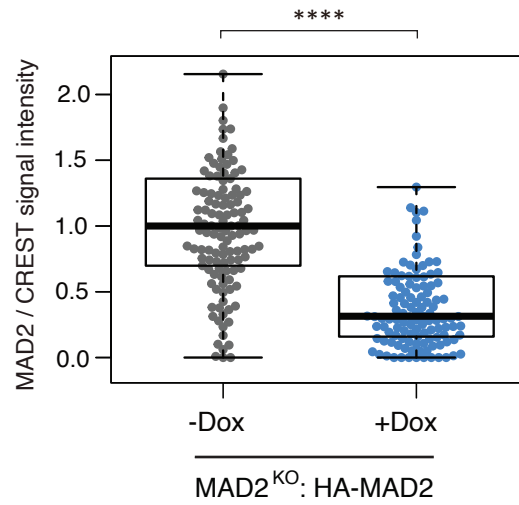

Supplement: Supplementary file 8 — Figure S7. The specificity of MAD2 antibodies [file 41388_2020_1187_MOESM8_ESM.pdf]
